# Adult presentation of symptomatic annular pancreas treated with gastrojejunostomy

**DOI:** 10.1093/jscr/rjae638

**Published:** 2024-10-15

**Authors:** Violet M Kryzsko, Maria E Tecos, Keely L Buesing, Reynold Henry

**Affiliations:** Division of Acute Care Surgery, University of Nebraska Medical Center, 620 S 42nd St, Omaha, NE 68105, United States; Division of Pediatric Surgery, Oregon Health & Science University, 3181 S.W. Sam Jackson Park Road, Portland, OR 97239, United States; Division of Acute Care Surgery, University of Nebraska Medical Center, 620 S 42nd St, Omaha, NE 68105, United States; Division of Acute Care Surgery, University of Nebraska Medical Center, 620 S 42nd St, Omaha, NE 68105, United States

**Keywords:** annular pancreas, duodenal obstruction, open gastrojejunostomy, adult bowel obstruction

## Abstract

Annular pancreas is a rare congenital abnormality that is often considered a neonatal condition, though in few cases it can present in adulthood. Patients diagnosed with annular pancreas in adulthood are often asymptomatic and diagnosed incidentally from radiologic studies. While there is no existing treatment protocol for symptomatic annular pancreas, multiple surgical approaches have been documented in the literature. In this case study we present a 49-year-old female patient who presented with gastric outlet obstruction secondary to annular pancreas. The patient was treated with nasogastric decompression and open side-to-side gastrojejunostomy with an antecolic approach to bypass the obstructed duodenal segment. The surgery was successful, followed by an uneventful postoperative course and discharge on postoperative Day 4. By 5-week outpatient follow-up the patient had resolution of her symptoms and achieved weight gain.

## Introduction

Annular pancreas is a rare congenital abnormality that arises due to failure of complete rotation of the ventral bud of the pancreas during fetal development. It is often considered a pediatric condition, with the incidence, clinical presentation, and treatment of annular pancreas in the neonatal population being well-described in the literature [1]. Most adult patients with annular pancreas are asymptomatic and are diagnosed after incidental imaging findings. When symptomatic, this condition commonly presents with abdominal pain, duodenal obstruction, and pancreatitis [2]. The prevalence of annular pancreas varies in the literature with discrepancies between rates found in autopsy reports and in data from endoscopic retrograde cholangiopancreatography (ERCP) studies [3]. A recent epidemiological study determined the prevalence of annular pancreas in the United States to be 3.4 per 100 000 population [4]. While adult presentation is not comparatively less likely based on epidemiological studies and literature review, symptomatic presentation is less specific and is challenging to diagnose.

## Case report

Our patient was a 49-year-old Caucasian female with a history of gastroesophageal reflux disease, duodenal bulb ulcer with stricture, anxiety, depression, migraines, and fibromyalgia, who presented to the emergency department with a 4-month history of severe epigastric abdominal pain, nausea, and vomiting, anorexia, and over 20-pound unintentional weight loss. She reported a history of only being able to eat very small meals. She had no prior history of pancreatitis. Surgical history was significant for prior cholecystectomy at age 36. Her medications included pantoprazole, sucralfate, metoclopramide, and dicyclomine which she had been taking as prescribed with minimal relief of her symptoms. She was hospitalized twice in the prior month for her symptoms and seen by a gastroenterologist in the outpatient setting. Diagnostic studies prior to hospital presentation included abdominal computed tomography (CT) scan with intravenous contrast, esophagogastroduodenoscopy (EGD), colonoscopy, and gastric emptying study, after which she received a diagnosis of gastroparesis. Her EGD was complicated by the presence of undigested food remaining in the stomach, and abdominal CT reading was reported as mild duodenal wall thickening that may represent duodenitis. At the time of presentation, she had not passed stool for 4 days.

**Figure 1:**
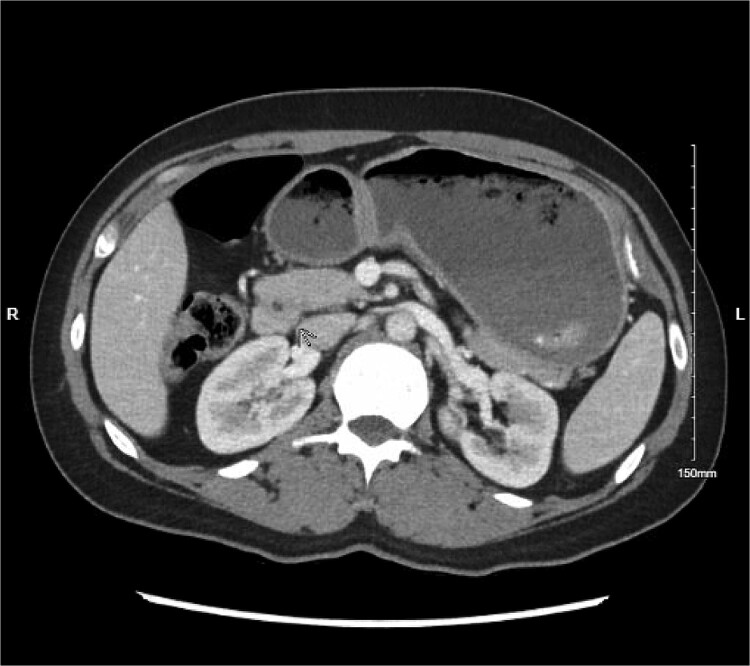
Abdominal CT with IV contrast showing fluid distension of the stomach and duodenal bulb with encirclement of pancreatic tissue around the first portion of the duodenum.

**Figure 2:**
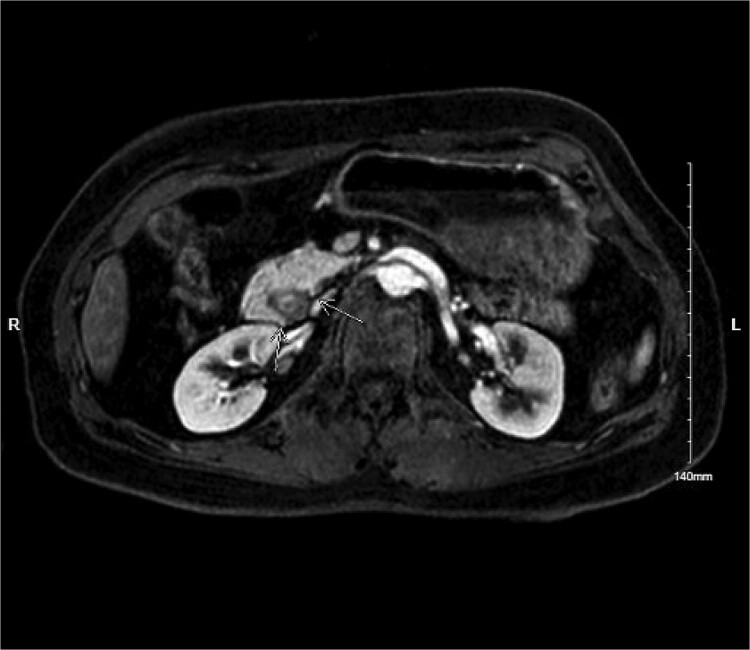
MRI MRCP showing annular pancreas causing near complete obstruction of the second portion of the duodenum.

Physical exam was significant for epigastric and bilateral upper quadrant abdominal tenderness without guarding. Notably, her urinalysis was positive for ketones. Abdominal CT with intravenous contrast ordered in the emergency department revealed a near complete annular pancreas at the level of the first portion of the duodenum with concurrent gastric and duodenal bulb distension with no evidence of pancreatitis (See Fig. 1). Nasogastric tube decompression and antiemetic therapy were initiated, and the patient underwent magnetic resonance cholangiopancreatography (MRCP), which revealed severe duodenal narrowing at the second portion with circumferential encasement of the pancreas (See Fig. 2).

Definitive surgical treatment with open gastrojejunostomy to bypass the obstruction was planned for hospital Day 3 after nasogastric tube decompression was achieved. An open side-to-side gastrojejunostomy using an antecolic approach was completed without complication. The postoperative hospital course was largely uneventful, and she was discharged on postoperative day 4 to home. At the clinic follow-up appointment 5 days after hospital discharge changes necessary in her medication regimen were identified. At the time of her second clinic follow up visit 5 weeks later, she had achieved weight gain, significant improvement in her oral intake of both solids and liquids, and resolution of her nausea and vomiting.

## Discussion

As annular pancreas is a rare congenital condition, it is often not considered on the differential diagnosis for gastric outlet obstruction in adults. Symptomatic annular pancreas in the adult population has nonspecific signs and symptoms, which renders diagnosis challenging for clinicians. Prompt recognition and diagnosis of annular pancreas is important, as it is known to have associations with pancreatic cancers, pancreatitis, biliary obstruction, and, in the case of this patient, peptic ulcer disease and gastric outlet obstruction. Quality of life can be significantly diminished for patients who do not receive sufficient or definitive treatment [1]. Imaging is critical to identification of annular pancreas; abdominal CT or magnetic resonance imaging that reveals pancreatic tissue posterolateral to the second portion of the duodenum is considered 92% sensitive and 100% specific for annular pancreas, regardless of the pancreatic tissue being circumferential around the duodenum [3].

Currently, there exists no treatment protocol for annular pancreas. Asymptomatic patients can be managed with conservative measures or no interventions at all. Surgical intervention must be individualized to the patient, their anatomy, and their presenting symptoms [4]. Open antecolic gastrojejunostomy was successful in the definitive treatment of this patient with duodenal obstruction. Duodenojejunostomy and duodenoduodenostomy are also reported in the literature, more commonly in pediatric cases. These options for surgical bypass of mechanical obstruction are preferred to excision of pancreatic ring tissue, as such operations have significant risk of complication and are not as effective in achieving cure of obstruction [1, 2].
